# Spanish is better than English for discriminating Portuguese vowels: acoustic similarity versus vowel inventory size

**DOI:** 10.3389/fpsyg.2014.01188

**Published:** 2014-10-29

**Authors:** Jaydene Elvin, Paola Escudero, Polina Vasiliev

**Affiliations:** ^1^The MARCS Institute, University of Western SydneySydney, NSW, Australia; ^2^Department of Spanish and Portuguese, University of California in Los AngelesLos Angeles, CA, USA

**Keywords:** non-native speech perception, acoustic similarity, vowel inventory, vowel discrimination, vowel perception

## Abstract

Second language (L2) learners often struggle to distinguish sound contrasts that are not present in their native language (L1). Models of non-native and L2 sound perception claim that perceptual similarity between L1 and L2 sound contrasts correctly predicts discrimination by naïve listeners and L2 learners. The present study tested the explanatory power of vowel inventory size versus acoustic properties as predictors of discrimination accuracy when naïve Australian English (AusE) and Iberian Spanish (IS) listeners are presented with six Brazilian Portuguese (BP) vowel contrasts. Our results show that IS listeners outperformed AusE listeners, confirming that cross-linguistic acoustic properties, rather than cross-linguistic vowel inventory sizes, successfully predict non-native discrimination difficulty. Furthermore, acoustic distance between BP vowels and closest L1 vowels successfully predicted differential levels of difficulty among the six BP contrasts, with BP /e-i/ and /o-u/ being the most difficult for both listener groups. We discuss the importance of our findings for the adequacy of models of L2 speech perception.

## INTRODUCTION

It is widely recognized that second language (L2) learners are often unable to distinguish sound contrasts that are not present in their native language (L1). A well-known example is the English /i-ɪ / vowel contrast which is discriminated poorly by listeners of many L1 backgrounds including Spanish (Fox et al., 1995; Flege et al., 1997; Escudero and Boersma, 2004; Escudero, 2005; Morrison, 2009), Mandarin (Flege et al., 1997), Portuguese (Rauber et al., 2005), and Russian (Kondaurova and Francis, 2008). However, not all contrasts that are absent in the L1 are equally difficult to discriminate. Models of non-native and L2 sound perception, such as the Second-Language Linguistic Perception Model (L2LP; Escudero, 2005, 2006, 2009) and the Perceptual Assimilation Model (PAM; Best, 1995) and its extension to L2 acquisition (PAM-L2; Best and Tyler, 2007) claim that perceptual similarity between native sounds and target language contrasts predicts how accurately naïve listeners and L2 learners will identify the members of those contrasts.

Both L2LP and PAM predict high difficulty in discrimination of target language contrasts that do not exist in the listener’s L1, which is commonly the case when the L1 has a smaller sound inventory than the L2. This results in many target language contrasts being assimilated to a single native category, which is known as single category assimilation in the PAM (e.g., Best, 1995; Levy, 2009) and as the new scenario in L2LP (e.g., Escudero, 2009, 2006). On the other hand, target language sounds that are mapped to two different native categories (PAM’s two-category assimilation and L2LP’s similar scenario) are less problematic for learners (e.g., Best et al., 1996; Escudero and Boersma, 2004). A third scenario, referred to as uncategorized assimilation in PAM and multiple category assimilation for L2LP, occurs when two L2 vowels in a binary contrast are perceived as belonging to more than two vowel categories in the L1 (Escudero and Boersma, 2002). This scenario usually occurs when the vowel inventory of the target language is smaller than that of the L1. Discrimination in this scenario is expected to be less problematic for learners than the case of single category assimilation (Escudero, 2005; Bohn et al., 2011). However, Escudero and Boersma (2002) suggest that multiple category assimilation may be problematic when it leads to a subset problem where the learner needs to realize on the basis of positive evidence alone that some features or vowels of their own language do not exist in the target language and may find it difficult not to perceive the extra L1 category.

The present study aims at testing the explanatory power of two possible predictors of non-native vowel discrimination accuracy, namely vowel inventory size versus vowel acoustic properties. To this end, we compare how naïve Australian English (AusE) and Iberian Spanish (IS) listeners discriminate vowels in Brazilian Portuguese (BP). These three languages were chosen because they have different vowel inventory sizes: IS has the smallest number of vowels with only five stressed monophthongs, /i, e, a, o, u/, BP has a slightly larger inventory of seven stressed oral monophthongs, /i, e, ε, a, o, ɔ, u/, and AusE has the largest vowel inventory with 12 monophthongs, /iː, ɪ, e, eː, ɜː, ɐ, ɐː, æ, o, ɔ, ʊ, ʉː/. The two listener groups were chosen because vowel inventory size is likely to determine the specific learning scenarios, from those mentioned above, that a listener will experience when confronted with a new language. Specifically, AusE listeners who have a large vowel inventory are likely to accurately discriminate most BP vowel contrasts, as they all exist in their L1. They may perceive some BP vowels as multiple AusE vowels but as mentioned above, substantial difficulty for this learning scenario is not expected. Conversely, Spanish learners who have a smaller vowel inventory will face single-category assimilation scenarios for BP /e-ε/ and /o-ɔ/ as these contrasts are not present in Spanish. Below we will review the evidence supporting vowel inventory size as a successful predictor of non-native and L2 discrimination accuracy, together with findings suggesting that a comparison of the acoustic properties of the listeners’ native vowels and those of the target language may be a better predictor.

To investigate the effect of L1 vowel inventory size on L2 perception, Scholes (1968) had six non-native speakers of English classify synthetic vowels sounds firstly in terms of their own native vowels and then in terms of English vowels. Scholes (1968) found that listeners’ categorization of the stimuli using English labels in the second condition was largely predictable by their L1 responses from the first condition. Fox et al. (1995) have interpreted Scholes (1968) findings as an indication that vowel identification depends in part on the number and nature of the listener’s native vowel categories. Bradlow (1995) also found that listeners’ categorization of Spanish /i-e/ and /o-u/ synthetic continua was strongly affected by the presence of extra AE categories. Fox et al. (1995) compared vowel perception of monolingual English speakers and Spanish bilinguals and found that English listeners use more phonetic features to distinguish vowels. Specifically, the authors showed that the structure of a listener’s vowel space is affected by their L1 native vowel inventory, as English listeners used three underlying dimensions (vowel height, vowel backness, and vowel centrality), whereas Spanish listeners used only two dimensions.

Other studies suggest that learners with a larger L1 vowel inventory than the target language should be better at learning new vowel categories than learners with smaller L1 vowel inventories than the target language. For example, Iverson and Evans (2007) found that when identifying English vowels, German and Norwegian listeners, who have a larger L1 vowel inventory than English, were more accurate than Spanish and French listeners, whose L1 vowel inventory is smaller than English, despite the fact that both groups used the same acoustic cues to identify the English vowels. In a more recent study, Iverson and Evans (2009) found more improvement for German than for Spanish listeners after auditory training with English vowels, which led the authors to conclude that having a larger and more complex vowel system (German) may facilitate vowel learning.

Based on the above findings supporting the predictive role of vowel inventory size in non-native perception (e.g., Scholes, 1968; Bradlow, 1995; Fox et al., 1995; Iverson and Evans, 2007, 2009), Spanish listeners should be less accurate at discriminating BP vowels than AusE listeners. As mentioned above, BP /e-ε/ and /o-ɔ/ should be most difficult as they are likely to be perceived as a single Spanish category, given that /ε/ and /ɔ/ are not present in Spanish. Previous studies have indeed shown that Spanish natives, including those who began learning the target language at an early age, have substantial difficulty perceiving the Catalan mid-vowel contrasts /e-ε/ and /o-ɔ/ (e.g., Pallier et al., 1997, 2001; Sebastián-Gallés and Soto-Faraco, 1999; Sebastián-Gallés et al., 2005; Mora et al., 2010). If vowel inventory size is a good predictor for non-native vowel perception, Spanish listeners should experience a similar level of difficulty with the same mid-vowel contrasts in BP.

On the other hand, AusE listeners should perform better overall than Spanish listeners due to their larger vowel inventory and they should experience fewer problems with BP mid-vowel contrasts as their larger vowel inventory contains similar contrasts, namely /e-εː/ and /o-ɔ/. Although little is known regarding AusE listeners’ perception of Portuguese vowels, a number of studies have examined American English (AE) learners’ perception of Portuguese vowels. For example, Díaz Granado (2011) observed that while L2 and L3 AE learners of BP had difficulties producing the BP /e-ε/ contrast, they were significantly better at discriminating BP /e-ε/ and at assimilating this BP contrast to the closest English contrast (as represented by the words “bait”-“bet”) than AE listeners who had no experience with BP. This finding indicates that unlike Spanish listeners, AE listeners’ initial difficulty with this contrast diminishes with experience, supporting the claim that a larger and more complex vowel inventory may facilitate vowel learning (Iverson and Evans, 2009). However, in contrast to the findings of Díaz Granado (2011), Vasiliev (2013) found that AE listeners from California with no experience with BP had hardly any difficulty with BP /e-ε/, as shown by a discrimination accuracy at above 90%, while they had considerable difficulty with BP /e-i/ and /o-u/ (accuracy between 60 and 70%). This finding suggests that examining differences in the number and type of vowel categories between the L1 and the target language may not be sufficient to fully account for differences in non-native perception.

Thus, it seems important to consider the role of acoustic properties in explaining findings such as those reported in Vasiliev (2013). Unlike PAM and PAM-L2, which rely on perceptual assimilation results to predict discrimination accuracy, the L2LP model (Escudero, 2005, 2009) explicitly proposes that non-native vowel discrimination can be reliably predicted with detailed acoustic comparisons of the target language and native sound categories. The model puts forward that the perception of native sounds is optimal because native listeners’ perception matches the specific acoustic properties of native sounds (Escudero, 2006, 2009; Escudero et al., 2014). Therefore, according to the L2LP model, a listener’s initial non-native sound perception should closely match the acoustic properties of sounds as they are produced in the listener’s L1 (Escudero and Boersma, 2004; Escudero, 2005; Escudero and Williams, 2012; Escudero et al., 2012, 2014). The model also advances that as a result of this direct link between production and perception, if languages or dialects differ in their productions of the same phonemes, those differences should be evident in cross-dialectal and cross-linguistic perception (Escudero and Boersma, 2004; Escudero, 2005).

The validity of this cross-linguistic and cross-dialectal proposal was first demonstrated empirically by the differential perception of the same tokens of /i/ and /ɪ / in native Standard Scottish English (SSE) and Standard Southern British English (SSBE) listeners (Escudero and Boersma, 2004), and in monolingual Peruvian Spanish (PS) listeners (Escudero, 2005). A growing body of recent studies (e.g., Escudero and Chládková, 2010; Escudero and Vasiliev, 2011; Escudero et al., 2014) further supports the L2LP proposal, demonstrating that the specific acoustic properties of a language or a particular dialect substantially affect non-native vowel perception. These findings are in contrast with a number of previous studies that have challenged the hypothesis that acoustic properties always predict native and non-native perception. For example, Strange et al. (2004) compared the acoustic properties of AE and Northern German (NG) vowels using linear discriminant analysis models and found that the models’ classifications did not accurately predict NG listeners’ perceptual assimilations of AE vowels and that the consonantal context in which NG vowels were produced did not affect AE listeners’ classifications, despite the fact that there were significant differences in acoustic properties of the NG vowel when produced in the different contexts.

Escudero and Vasiliev (2011) directly tested Strange et al.’s (2004) context-independent hypothesis on PS perception of Canadian English (CE) and Canadian French (CF) /ε/ and /æ/ and found that context-specific acoustic differences in the production of the two sounds between CE and CF resulted in differences in PS listeners’ assimilation of these phones to native categories. Furthermore, linear discriminant analysis revealed that acoustic similarity between native and target language vowels was a very good predictor of context-specific perceptual mappings. Discriminant analyses including native and target language vowel acoustics have also been shown to successfully predict assimilation patterns for Russian listeners of AE (Gilichinskaya and Strange, 2010) and differences in L2 (English) vowel perception due to dialectal differences in native (Dutch) vowel productions by North Holland versus Flanders speakers (Escudero et al., 2012).

Recent studies have shown that cross-linguistic acoustic similarity can successfully predict difficulty in non-native vowel perception for one group of English listeners (Vasiliev, 2013) and for two groups of listeners with Spanish and Italian as L1s (Escudero et al., 2014). Given that the present study compares two listener groups, the findings of Escudero et al. (2014) are particularly relevant. Their acoustic analyses predicted different perceptual difficulties for the categorization of Southern British English vowels despite the fact that the two listener groups (Salento Italian and Peruvian Spanish) shared the same phonemic inventory of five vowels. This finding suggests that even when languages have the same vowel inventories, cross-language acoustic similarity has an important role in predicting L2 perceptual difficulty, as only acoustics predicted the observed differences in non-native vowel perception between the two listener groups. We therefore also examined the explanatory power of a comparison of vowel acoustic properties for predicting IS and AusE listeners’ discrimination accuracy of BP contrasts.

**FIGURE 1:**
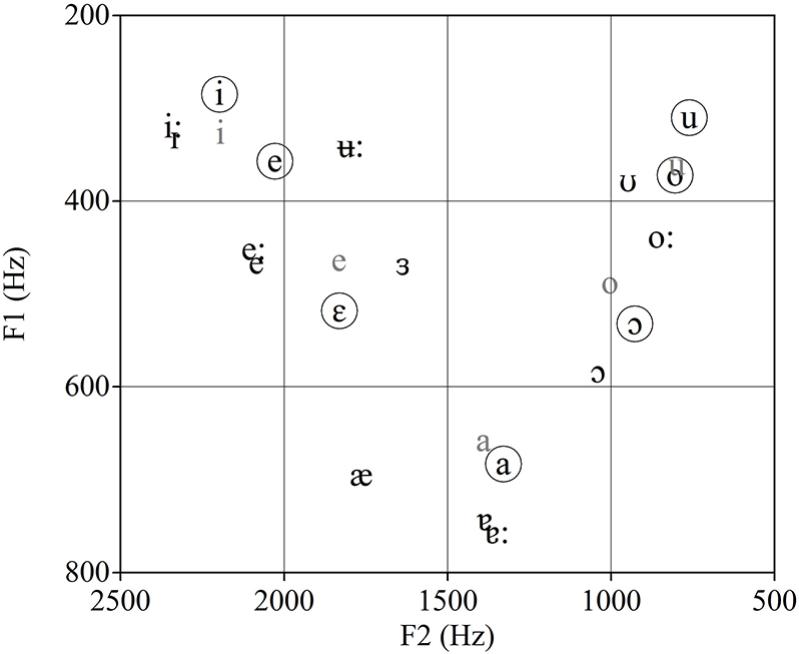
**Male speakers’ average F1 and F2 values for Brazilian Portuguese (BP; black with circles), Australian English (AusE; black), and Iberian Spanish (IS; gray)**.

**Figure 1** shows the F1 and F2 values of the seven vowels of BP (Escudero et al., 2009b), together with the five vowels of IS (Chládková et al., 2011) and the 12 AusE monopthongs (Cox, 2006). Although AusE has a larger vowel inventory than BP as well as contrasts that may be comparable to the BP contrasts /e-ε/ and /o-ɔ/ that are not present in IS, visual inspection of **Figure 1** shows that AusE and IS vowels compare similarly to BP vowels, which would predict similar non-native vowel discrimination across these two listener groups. For example, the BP contrasts /e-i/ and /o-u/ should be more problematic for both IS and AusE listeners than the other four contrasts as a result of single-category assimilation for IS listeners and single and multiple category assimilation for AusE listeners, while the other four contrasts have vowels that visually appear closest to two different native vowels. That is, both BP /o/ and /u/ are acoustically close to one native category /u/ for IS, and /ʊ/ for AusE. In the case of the BP /e/ and /i/ both vowels seem to be acoustically close to one native IS category /i/, yet multiple categories (/iː / and /ɪ /) for AusE.

While plotting the vowels of each language acoustically provides insight for cross-linguistic differences in the location of vowels within the F1–F2 acoustic space, the calculation of the Euclidean Distances (ED)^1^ between target vowel contrasts (BP) and native (IS or AusE) vowels can be used as a quantitative measure of cross-linguistic similarity. **Table 1** shows the ED between the six BP vowel contrasts considered in this study and the first and second acoustically closest IS or AusE vowel as well as the difference in ED between the first and second acoustically closest vowels. For all BP contrasts, the two vowels involved are acoustically closer to an IS than to an AusE vowel, as shown by the smaller EDs. Additionally, the differences in ED between the first and second acoustically closest vowels are much smaller for AusE than for IS, which suggests that this second native category is a likely choice for AusE but not for IS listeners. Thus, an acoustic comparison predicts overall higher accuracy for IS than AusE listeners. This is because a single IS vowel is acoustically similar to a corresponding BP vowel, while for AusE at least two competing native vowels are in close proximity (neither of which is as close to the BP vowel as the closest IS vowel), which may at least slow discrimination and even lead to confusion.

**Table 1:** Euclidean distances (ED) between the acoustic closest (first) and second closest (second) native vowel (IS and AusE) and each of the two vowels in the six BP contrasts as well as the difference in ED between the first and second closest native vowels.

BP vowel	IS: first/second closest vowel						AusE: first/second closest vowel					
/A-B/	to A	ED	ED_diff	to B	ED	ED_diff	to A	ED	ED_diff	to B	ED	ED_diff
/a-ɔ/	a/o	0.34/2.37	2.03	o/a	0.60/2.71	2.11	ɐ/ɐː	0.52/0.55	0.03	ɔ/oː	0.81/0.97	0.16
/a-ε/	a/o	0.34/2.37	2.03	e/a	0.47/2.15	1.68	ɐ/ɐː	0.52/0.55	0.03	ɜː/e	0.87/0.98	0.11
/e-i/	i/e	0.61/1.21	0.6	i/e	0.43/2.12	1.69	eː/ɪ	0.92/0.98	0.06	iː/ɪ	0.55/0.63	0.08
/o-u/	u/o	0.11/1.66	0.55	u/o	0.57/2.3	1.73	oː/ʊ	0.69/0.94	0.25	oː/ʊ	1.38/1.41	0.03
/e-ε/	i/e	0.61/1.21	0.6	e/a	0.47/2.15	1.68	eː/ɪ	0.92/0.98	0.06	ɜː/e	0.87/0.98	0.11
/o-ɔ/	u/o	0.11/1.66	0.55	o/u	0.60/1.77	1.17	oː/ʊ	0.69/0.94	0.25	ɔ/oː	0.81/0.97	0.16

The EDs reported in **Table 1** also support the predictions based on **Figure 1** regarding the relative discrimination difficulty of BP contrasts and are in line with Vasiliev (2013) findings for AE listeners. For IS, the EDs confirm that IS /i/ is acoustically the closet vowel to BP /e/ and /i/ and that IS /u/ is acoustically close to both BP /o/ and /u/, which will lead to discrimination difficulty as a result of single-category assimilation. For AusE listeners, difficulty in discrimination is also predicted when there is a neutralization of a L2 contrast caused by multiple category assimilation. That is, two target language vowels are each acoustically close to the same two or more L1 vowels, resulting in a partial or total acoustic overlap. For instance, although **Figure 1** suggests that only AusE /ʊ/ is acoustically close to both BP /o/ and /u/, the values presented in **Table 1** show that in addition to /ʊ/, AusE /oː/ is also acoustically close to the two BP vowels, resulting in a total acoustic overlap for BP /o-u/, which will lead to difficulty in discrimination. For BP /e/ and /i/, at first inspection of the EDs, it may seem that the closest AusE vowels are /eː/ and /iː/ respectively, suggesting possible two-category assimilation and no difficulty in discrimination. However, the second closest AusE vowel to both BP /e/ and /i/ is AusE /ɪ/, which, due to its acoustic proximity, may well be a competing attractor for BP /e/ and /i/, suggesting a partial acoustic overlap which could lead to confusion and discrimination difficulty for this contrast. Conversely, multiple category assimilation is unlikely to be problematic for AusE listeners in cases like the BP /a-ε/ contrast where no acoustic overlap occurs.

In sum, if vowel inventory size is a good predictor of non-native vowel discrimination, AusE listeners should be more accurate at discriminating BP vowels than IS listeners because the probability of having vowels which are phonetically similar to the BP vowel system is higher for speakers of larger vowel inventories than speakers of smaller vowel inventories. In particular, the BP contrasts /e-ε/ and /o-ɔ/ should be more difficult to discriminate for IS than AusE listeners, as the lack of /ε/ and /ɔ/ in Spanish may result in single-category assimilation. Alternatively, if acoustic similarity measures (as shown in **Figure 1**; **Table 1**) determine success in non-native vowel discrimination, following the L2LP model’s acoustic hypothesis, both groups should find the same vowel contrasts equally difficult or easy to discriminate and that in particular, the BP /e-i/ and /o-u/ contrasts should be most difficult for both groups. Furthermore, if acoustic values are a good predictor of non-native discrimination accuracy IS listeners should be overall more accurate in discriminating BP vowels than AusE listeners.

## MATERIALS AND METHODS

### PARTICIPANTS

Listeners were 16 AusE and 15 IS functional monolinguals aged between 19 and 55 (mean age, 25.8 for AusE and 25.9 for IS). The AusE participants were tested at the University of Western Sydney and reported little to very basic knowledge of any foreign language and no knowledge of Portuguese. The IS participants were all tested at the Universidad Complutense and at the Universidad Nacional de Educación a Distancia, both in Madrid. They reported a basic to intermediate knowledge of English but did not use English in their daily lives. They also reported very little knowledge of another foreign language and no knowledge of Portuguese, which suggests that they are functional monolinguals. All participants provided informed consent in accordance with the ethical protocols in place at the Universidad Nacional de Educacin a Distancia and the University of Western Sydney Human Research Ethics Committee.

### STIMULI

**FIGURE 2:**
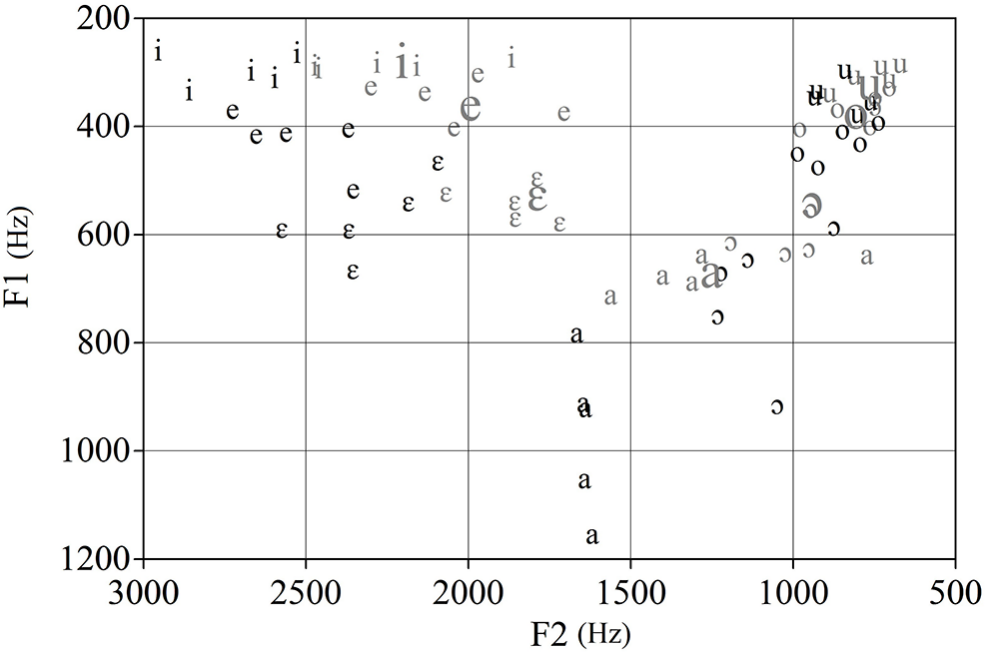
**F1 and F2 values for the male (gray, small font) and female (black, small font) natural BP vowel tokens, and for the synthetic vowel tokens (gray, large font)**.

Listeners were presented with 70 BP isolated vowel tokens produced by five male and five female monolingual speakers of BP from Sao Paulo, which were selected from a larger corpus reported in Escudero et al. (2009b). The seven BP isolated vowel (V) tokens (i, e, ε, a, o, ɔ, u), were extracted from nonce words in the /fVfe/ context produced in a carrier phrase. We also used seven synthetic tokens representing each of the seven BP vowels for the A and B stimuli in the XAB categorical discrimination task that will be described below. These tokens were synthesized using the computer program Praat (Boersma and Weenink, 1992–2014) and were based on the average F1 and F2 values for BP vowels shown in **Figure 1**. **Figure 2** shows the male and female F1 and F2 values for the natural vowel tokens in relation to the synthesized BP prototypes.

### PROCEDURE

Participants were tested in a sound-attenuated room in Sydney and in a sound-proof booth in Madrid. Following the same procedure as Escudero et al. (2009a), Escudero and Wanrooij (2010), and Escudero and Williams (2012), participants were presented with an auditory discrimination task in the XAB format, which was run on a laptop computer using Praat. Testing consisted of six categorial discrimination tasks, with each task containing one of six BP contrasts, /a-ɔ/, /a-ε/, /e-i/, /o-u/, /e-ε/, and /o-ɔ/. In each trial, listeners were presented with three vowel tokens, one after the other, and were asked to decide whether the first vowel (X) sounded more like the second (A) or the third (B) by clicking with a mouse on the corresponding options (either “2” or “3”) on the screen. There were 44 trials for each contrast, and in each trial, the order for the A and B response was counterbalanced, namely XAB and XBA. The X sounds were the natural tokens and the A and B responses were always the two synthetic tokens described above, which mimic the acoustic properties of the specific BP vowels, involved in each of the six XAB tasks. One synthetic token of each of the two vowels was presented twice as the X stimulus to ensure that listeners understood the task and were able to match acoustically equal tokens.

We used synthetic stimuli with mean values of naturally produced BP vowels (from Escudero et al., 2009b) in order for listeners to make their discrimination decision based on a comparison between individual tokens and average or prototypical values. This results in a categorical discrimination task, where listeners are expected to base their decision of whether A and B are more similar to X on phonemic rather than acoustic differences, as they have to compare different types of stimuli (synthetic versus natural) with different acoustic properties (individual tokens versus average values). The phonemic nature of this XAB task is further strengthened with the use of an inter-stimulus interval (ISI) of 1.2 s to ensure language-specific phonological processing (Escudero et al., 2009a). Our task, stimuli types and ISI are identical to those of previous studies which have successfully shown differences between native and non-native listeners for specific vowel contrasts (e.g., Escudero et al., 2009a, 2011; Escudero and Wanrooij, 2010; Escudero and Williams, 2012, 2014). These studies have also shown that this task avoids listeners’ reliance on native orthography, which has been shown to affect their non-native perception.

Oral instructions were given in the listeners’ L1 (English or Spanish). As in Escudero and Wanrooij (2010), a practice session was conducted using a fairly easy contrast, namely /i-u/. The experiment took approximately 30 min to complete as listeners took around 5 min to complete each individual XAB task.

## RESULTS

**Table 2** shows the percentage correct with which the AusE and IS monolingual listeners discriminated the BP vowel contrasts.

**Table 2:** AusE and IS monolingual listeners’ accuracy scores for the 6 BP contrasts.

	/a-ɔ/	/a-ε/	/e-i/	/o-u/	/e-ε/	/o-ɔ/
IS	83.18 SE: 2.89 L: 77.25 U: 89.08	98.5 2.03 94.36 102.64	73.67 2.71 68.13 79.2	64.87 3.24 58.15 71.58	82.5 2.99 76.38 88.62	89.33 2.3 84.63 94.04
AusE	75.63 SE: 2.80 L: 69.90 U: 81.35	92.19 1.96 88.18 96.2	66.25 2.62 60.89 71.61	65.94 3.18 59.43 72.44	82.81 2.9 76.88 88.74	80.31 2.23 75.76 84.87

*SE and Lower (L) and Upper (U) bound confidence intervals of the means are also given.*

The table shows that, with the exception of BP /o-u/ and /e-ε/, IS listeners had higher discrimination accuracy than AusE listeners. A repeated measures ANOVA with group as a between-subjects factor and contrast as a within-subjects factor revealed main effects of group [*F*(1,29) = 5.457, *p* = 0.027, $\eta_{p}^{2}$ = 0.158) and contrast [*F*(5,80) = 37.764, *p* = < 0.001, $\eta_{p}^{2}$ = 0.566], but no interaction between contrast and ^∗^listener group [*F*(5,80) = 1.550, *p* = 0.178, $\eta_{p}^{2}$ = 0.051]. This indicates that both groups found the same contrasts equally easy or difficult, but that IS listeners had higher accuracy overall.

To compare accuracy across BP contrasts, paired samples *t*-tests including all listeners pooled together were conducted for each possible comparison of the six contrasts, with α = 0.0033 (15 comparisons). The results indicated that /e-i/ and /o-u/ had significantly lower accuracy than the remaining five contrasts [*t*s(30) = 7.705–12.805, all *p*s(two-tailed) < 0.001], indicating that they were the most difficult to discriminate. The paired *t*-test that compared accuracy for /e-i/ and /o-u/ did not yield significance [*t*(30) = 1.583, *p*(two-tailed) = 0.124], indicating that these two contrasts were equally difficult. Finally, /a-ɔ/, /e-ε/ and /o-ɔ/ had comparable accuracy [*t*s(30) = 0.803–2.204, *p*s(two-tailed) = 0.035–0.428], but were more difficult than /a-ε/ [*t*s(30) = 5.890–8.163, all *p*s(two-tailed) < 0.001]. Following Escudero and Wanrooij (2010), the ranking of difficulty for both listener groups, ranging from the most to the least difficult BP vowel contrast, is as follows: /o-u/ ∼ /e-i/ > /a-ɔ/ ∼ /e-ε/ ∼ /o-ɔ/ > /a-ε/, where “∼” means equal or comparable difficulty and “>” means higher difficulty.

## DISCUSSION

The present study tested the explanatory power of two possible predictors for non-native discrimination difficulty, namely vowel inventory size versus a detailed comparison of acoustic properties across native and non-L1s. To this end, the discrimination of BP vowels by AusE and IS listeners was compared. Following predictions based on vowel inventory sizes, AusE listeners, whose native vowel system includes all of the BP contrasts, should outperform IS listeners, who only have five native vowels and lack two of the mid-vowels (/ε/ and /ɔ/) present in BP, which should result in single-category assimilation and poor discrimination. Alternatively, following the L2LP model’s acoustic hypothesis, if a comparison of vowel acoustic properties (see **Figure 1**; **Table 1**) successfully predicts non-native vowel discrimination, IS listeners should have higher accuracy overall in the discrimination of BP vowels. Acoustic properties also predict that both groups will have the same level of difficulty for all contrasts.

The findings are in line with the L2LP model’s acoustic hypothesis and the corresponding predictions based on the detailed comparison of BP, IS, and AusE vowels that was presented in the Introduction. That is, IS listeners did have higher overall accuracy than AusE listeners, and relative ease or difficulty for each BP contrast for both groups was largely predictable based on the acoustic comparisons presented in the Introduction (see **Figure 1**; **Table 1**). In particular, the BP contrasts /e-i/ and /o-u/ were indeed the most difficult, and /a-ε/ was the easiest to discriminate for both groups. It is interesting to note that unlike previous studies of Catalan, which shares a similar vowel inventory to BP, the mid-vowel contrasts /e-ε/ and /o-ɔ/ were not as difficult as the high-vowel contrasts /e-i/ and /o-u/ for IS listeners. Likewise, the findings of the present study were not in line with those of Díaz Granado (2011), yet they were comparable to those for Californian English (CE) listeners in Vasiliev (2013), as CE listeners also found /e-i/ and /o-u/ to be substantially difficult. However, unlike AusE listeners, CE listeners found /a-ɔ/ as difficult as /o-u/, and had significantly lower accuracy scores for /a-ɔ/ than for /e-ε/ and /o-ɔ/. As shown in Williams and Escudero (2014), differences in non-native vowel perception between native listeners with different English dialects are also explained by dialectal differences in English vowel production. In that respect, ongoing research comparing AusE, CE, and native BP listeners suggest that acoustic properties may be at the heart of the differential non-native patterns. This new study also demonstrates the validity of the BP stimuli used in the present study, as native BP listeners tested in Sao Paulo, Brazil, had accuracy scores of above 83% for the six BP vowel contrasts. Interestingly, a preliminary analysis also shows different levels of accuracy across vowel contrasts and that acoustic proximity is likely to explain this variability in native vowel perception.

The fact that IS listeners had overall higher accuracy despite their vowel inventory lacking the same contrasts that are present in both BP and AusE seems to suggest that vowels which are acoustically closer to the target vowels with no activation of competing categories are easier to discriminate. According to the acoustic predictions described in the Introduction, AusE listeners may use all the vowel categories that are acoustically close to the target BP vowel. This is likely to cause confusion because of the multiplicity of possible response options, resulting in the poorer performance shown in the present study. In other words, our acoustic predictions and discrimination findings seem to suggest that the number of mental representations (i.e., vowel categories) available to the listener influences native and non-native vowel perception. Further evidence for this claim was provided by Benders et al. (2012), who investigated the influence of stimulus range (i.e., different subsets of the Spanish /i-e/ continuum) and the number of available response categories on vowel categorization. The authors investigated the influence of the number of response categories by giving half of the participants /i/ and /e/ as responses and the other half, /i/, /e/, /a/, /o/, and /u/. The results showed that listeners who only chose from two response categories were more sensitive to broad and local acoustic contexts than listeners presented with five response categories. Listeners with only two response options were able to shift their boundary between /i/ and /e/ early, while listeners with five responses required more time. The authors argued that the delay in the boundary shift was caused by the availability of extra response options, causing them to be less precise in their responses (Benders et al., 2012). Although the participants were listening to their own L1, not all tokens presented were native-like, as they were part of a continuum, and so it seems that having more response options or a larger vowel inventory with more mental representations to choose from may result in difficulty in both native and non-native vowel perception.

If the number of mental representations is affecting the AusE listeners’ overall performance in discriminating BP vowels, this may indeed suggest an effect of multiple category assimilation, which can be problematic in vowel discrimination, as demonstrated with Dutch learners of Spanish whose multiple category assimilation patterns were reflected in their poorer classification of Spanish front vowels (Escudero and Boersma, 2002). Following from our acoustic comparisons, it may be that the AusE listeners’ lower overall discrimination scores are a result of multiple category assimilation affecting how well they discriminate BP contrasts. Recall from the values in **Table 1** that the difference in ED between the first and second acoustically closest vowels are much smaller for AusE than for IS, which suggests that this second native category is a likely choice for AusE but not for IS listeners. Furthermore, we predicted that discrimination would be difficult when an acoustic overlap (partial or total) was involved. Therefore the AusE listeners’ overall lower accuracy scores could be explained by these smaller differences in ED between the first and second acoustically closest vowels.

In order to test whether multiple category assimilation is a factor contributing to the overall lower discrimination accuracy by AusE listeners, we used general linear mixed modeling (run in R version 3.1.1) with the difference in ED between the vowel category of the X stimulus (the BP vowel category in that contrast) and the first and second closest native vowel (referred to as ΔED) as a predictor. We thus fit a binomial mixed model to our accuracy data using the *glmer* function (binomial family). We predicted that the smaller the ΔED (which results in multiple category assimilation), the lower the discrimination accuracy for that particular trial. ΔED was included as a fixed effect and participant and speaker as random effects (both slopes and intercepts). The model confirmed that ΔED predicted discrimination accuracy (β = 0.4395, SE = 0.1989, *z* = 2.210, *p* = 0.0271), with the positive β coefficient indicating that the larger the ΔED, the higher the accuracy. We therefore conclude that ΔED can account for the overall lower performance by the AusE participants, as a smaller ΔED is representative of multiple category assimilation and the resulting lower discrimination accuracy^2^. However, this is only for contrasts that result in complete or partial neutralization in non-native perception (e.g., for /e-i/ and /o-u/), whereas for contrasts involving MCA, but no neutralization (e.g., /a-ε/), no difficulty in discrimination is found for either listener group.

In sum, the present study demonstrates that vowel inventory size (even when acoustic similarity is assumed) may not be sufficient for accurately predicting L2 discrimination difficulty unless detailed acoustic comparisons (e.g., ED’s) are made as these comparisons yield more successful predictions (as previously shown in Escudero and Chládková, 2010; Escudero and Vasiliev, 2011; Escudero and Williams, 2011, 2012; Escudero et al., 2012, 2014). Despite differences in vowel inventory size, which would predict more success for AusE listeners, IS were overall more accurate at discriminating BP vowel contrasts than AusE listeners, with both groups finding the same BP contrasts equally difficult or easy to discriminate, as predicted by the acoustic proximity of IS to BP vowels. Ongoing research aims at demonstrating whether acoustic properties, vowel inventory or a combination of both explains different levels of discrimination for BP vowel contrasts across listeners from different English dialects. Furthermore, as the present study is only applicable to vowels, future research is necessary for testing whether the L2LP acoustic hypothesis could also be applied to predicting difficulty for L2 consonants.

## Conflict of Interest Statement

The authors declare that the research was conducted in the absence of any commercial or financial relationships that could be construed as a potential conflict of interest.

## Footnotes

1 F1 and F2 values for both BP and L1 vowels were used for computing Euclidean distances. The following equation was used to measure the distance in Bark between the two vowels: *d*(p,q) = √[(p1–q1)ˆ2+(?p2–q2)?ˆ2] or *d*(TV,L1v) = √[(TVF1–L1v1)ˆ2 + (?TVF2–L1vF2)?ˆ2], where d stands for Euclidean distance, TV for target vowel, L1v for native vowel, and F1 and F2 for this vowel’s average F1 and F2 values.

2 A second model including both ΔED and Contrasts showed that these two factors are even better predictors of discrimination accuracy, suggesting that ΔED can also explain the difference in difficulty across the different BP vowel contrasts.
